# Supplementary material for: Synchronization in time-varying random networks with vanishing connectivity
Source: Sci Rep. 2019 Jul 15;9:10207. doi: 10.1038/s41598-019-46345-y (PMC6629696; doi:10.1038/s41598-019-46345-y)
Supplement: Supplementary file 1 — Supplementary information [file 41598_2019_46345_MOESM1_ESM.pdf]

Synchronization in time-varying random networks with vanishing connectivity
Supplementary information

Marco Faggian[1,2], Francesco Ginelli[1], Fernando Rosas[3,4], and Zoran Levnajić[2,*]

[1]SUPA, Physics Department and ICSMB, King's College, University of Aberdeen, AB24 3UE (UK)
[2]Faculty of Information Studies in Novo Mesto, 8000 Novo Mesto (Slovenia)
[3]Centre of Complexity Science and Department of Mathematics, Imperial College London (UK)
[4]Department of Electrical and Electronic Engineering, Imperial College London (UK)

A Instantaneous topology in the low connectivity regime

We discuss the instantaneous topology in the low connectivity regime, showing that it is largely dominated by isolated single nodes and isolated single connected pairs.

Consider a node j going through a rewiring process: it disconnects all its links and attempts to forge new ones with probability $p = q/N$. For small connectivity q, the dominant result will be no links at all, with probability P_0 given by (for large enough N)

$$P_0 = \left(1 - \frac{q}{N}\right)^{N-1} \approx e^{-q}\,. \tag{1}$$

On the other hand, the node will establish a single link with probability

$$P_1 = \frac{q}{N}\left(1 - \frac{q}{N}\right)^{N-2} \approx \frac{q}{N}\,e^{-q}\,. \tag{2}$$

More generally, we can write the probability P_n of establishing n links (with $n \ll N$) as

$$P_n \approx \left(\frac{q}{N}\right)^n e^{-q}\,. \tag{3}$$

In order to conclude our reasoning, we now consider the case where the rewiring node establishes a single link. Prior to being linked with j, the receiving node itself will indeed be isolated with probability P_0 or it will have n links with probability P_n. Summing up, we easily see that – after a rewiring event – isolated nodes show up with probability P_0, while isolated couples connected by one single link appears with probability Q_1

$$Q_1 = P_0 P_1 \approx \frac{q}{N}\,e^{-2q}\,. \tag{4}$$

Higher order connected clusters with k >1 links in total are suppressed by a factor $(q/N)^k$ in their probability, that is

$$Q_k \approx \left(\frac{q}{N}\right)^k e^{-2q}\,. \tag{5}$$

They are thus by far less probable to appear than isolated nodes or isolated pairs of mutually coupled nodes.

B Local synchronization timescale

We finally discuss in details the solution of Eq. (6) of the main text

$$\delta\dot{\varphi} = \delta\omega - 2\varepsilon \sin\delta\varphi \,. \tag{6}$$

For $\delta\omega \neq 0$ the solution of Eq. (6) is given by

$$\delta\varphi(t) = 2\arctan\left[\frac{2\varepsilon}{\delta\omega}\left(1-\Omega\tanh\left[\varepsilon\Omega\left(t+C\right)\right]\right)\right] \tag{7}$$

with C being an integration constant fixed by initial conditions and

$$\Omega = \sqrt{1-\frac{\delta\omega^2}{4\varepsilon^2}} \approx 1-\frac{\delta\omega^2}{8\varepsilon^2} \tag{8}$$

in the strong coupling regime $\varepsilon \gg \delta\omega$.
In the large time limit $t \gg 1$ we also get

$$\delta\varphi(t) \to 2\arctan\left[\frac{2\varepsilon}{\delta\omega}\left(1-\Omega\right)\right] = \arcsin\left(\frac{\delta\omega}{2\varepsilon}\right) \tag{9}$$

where in the last equality we have made use of trigonometric identities to highlight the obvious form for the equilibrium constant solution of Eq. (6).

In the strong coupling limit we get readily the asymptotic solution quoted in the main text,

$$\delta\varphi(t) \approx \frac{\delta\omega}{2\varepsilon} \,. \tag{10}$$

The convergence to this solution is indeed controlled by the hyperbolic tangent term appearing in Eq. (35) of the main text, which for large times exponentially converges to unity. To be more precise, for large times one has

$$\tanh\left[\varepsilon\Omega\left(t+C\right)\right] \approx 1-2e^{-2\varepsilon\Omega t} \tag{11}$$

with the convergence timescale $(2\varepsilon\Omega)^{-1} \approx (2\varepsilon)^{-1}$.

In the special case $\delta\omega = 0$ the solution of Eq. (6) finally reads

$$\delta\varphi(t) = 2\arctan\left[De^{-2\varepsilon t}\right] \tag{12}$$

with D an integration constant. In the large time limit

$$\delta\varphi(t) \to 0 \tag{13}$$

exponentially fast with rate 2ε.
